# Nutritional characteristics of *Stereospermum chelonoides* (L.f.) DC., an underutilized edible wild fruit of dietary interest

**DOI:** 10.1016/j.heliyon.2024.e24193

**Published:** 2024-01-11

**Authors:** Mridul Kant Chaudhary, Deepali Tripathi, Ankita Misra, Satyendra Pratap Singh, Pankaj Kumar Srivastava, Vartika Gupta, Rabinarayan Acharya, Sharad Srivastava

**Affiliations:** a Pharmacognosy Division, CSIR-National Botanical Research Institute, Lucknow, U.P., 226001, India; b Environmental Technologies Division & ENVIS – NBRI, CSIR-National Botanical Research Institute, Lucknow, U.P., 226001, India; c CCRAS, New Delhi, 110058, India; d FEST Division, CSIR-Indian Institute of Toxicology Research, Lucknow, U.P., 226001, India; e Department of Postharvest Science, Agricultural Research Organization, Volcani Center, P.O.B 15159, HaMaccabim Road 68, Rishon LeZion, 7505101, Israel

**Keywords:** Wild fruit, *Stereospermum chelonoides*, Nutritional analysis, Patala, RP-HPLC-PDA, Antioxidant activity

## Abstract

Malnutrition and hunger is a serious global issue, however, wild fruits possess the potential of combatting it being rich in nutrients. *Stereospermum chelonoides* (L.f.) DC., commonly known as “Patala” in Ayurvedic text, is a large wild tree bearing edible, yet, underutilized fruits consumed by the locals in Western parts of India and neighboring countries. The present study focuses on the nutritional profile of *S. chelonoides* fruit along with quantification of bioactive constituents using RP-HPLC-PDA and evaluation of *in-vitro* anti-oxidant and, anti-microbial activity. The fruit was found rich in nutritional composition having protein (2.41 % ± 0.007), fibre (3.46 % ± 0.02) and carbohydrate (90.19 % ± 1.73) with energy value of 368.2 ± 3.94 Kcal/100g. The elemental analysis of fruit resulted in macronutrients Ca, Mg and Na and micronutrients Fe, Mn, Zn, and Cu in amounts comparable to common marketed fruits. The RP-HPLC-PDA analysis revealed the presence of six phenolic compounds in all 3 extracts made from the fruit in which highest amount are present in hydro-alcoholic extract. All the extracts exhibited potent antioxidant activity evaluated through DPPH assay and oxygen radical absorbing capacity (ORAC), with highest activity in hydro-alcoholic extract. All the analyzed extracts also exhibited potent inhibition, against four human pathogens namely *Pseudomonas aeruginosa*, *Vibrio cholerae*, *Escherichia coli,* and *Shigella flexneri*. Therefore, it is evident from the study that the fruit of *S. chelonoides* has immense potential as a nutraceutical supplement and may help in the management of nutrient deficiency and malnutrition among rural and tribal communities.

## 1 Introduction

Globally, about 828 million populations are suffering from hunger and around 3 billion people are undernourished [1]. In the Indian sub-continent, nutrient deficiency and malnutrition are quite prevalent. Statistics shows in India, 18.7 % of women (15–49 years) are malnutritioned and about 32.1 % of children (under 5 years) are underweight [2]. This may be due to the consumption of cereals and pulses as a staple food, whereas fruit and vegetables account for merely 9 % of the total calorie intake [3]. The predominant consumption of traditional starchy crops (cereals and root crops), especially by the rural communities creates micronutrient and protein malnutrition [4].

The food and agricultural organization of the United Nations (FAO) endorses the sustainable use of natural resources like wild fruits for nutrition as a means to promote dietary diversity [5]. Wild fruits are an integral part of the global food basket [6], with rich source of nutrients such as dietary fiber, protein, minerals, sugars, and health-promoting phytochemicals, essential for human consumption [4]. There is a need to educate people about the nutritional and health benefits of these wild fruits to promote their commercial utilization after scientific validation of their therapeutic/medicinal benefits.

*Stereospermum chelonoides* (L.f.) DC. synonym *Stereospermum suaveolens* (Roxb.) DC. (Bignoniaceae) commonly known as “Patala” in Ayurveda, is a large deciduous tree widely distributed in Indian sub-continent namely in central India, sub-Himalayan tract, western Peninsula, Myanmar and Bangladesh [7]. The fruit of *Patala* is eaten as vegetable by locals in Western states of India [8] and in countries of East Indies [9]. Apart from dietary uses, the plant is attributed to have tremendous medicinal properties. The fruits are used in hiccups and blood diseases [10]. Its root is one of the ingredient of classical Ayurvedic preparation *Dashamularishta* [11]. Decoction of Bark, root and flowers are used for the treatment of sore throat, bleeding diseases, asthma and as cooling drink in fever [12]. Owing to its ethnopharmacological uses in food and as medicine draws attention, the present study was designed to evaluate the nutritional profile of *S. chelonoides* fruit and quantification of bioactive constituents using RP-HPLC-PDA. Further, the radical scavenging and anti-microbial activity were validated through *in-vitro* assays. The nutritional profile of *S. chelonoides* fruit will promote its consumption and helps in management of nutrient deficiency and malnutrition among rural and tribal communities.

## 2 Materials and methods

### 2.1 Chemical and reagents

Gallic acid (>99 %), quercetin (>99 %), protocatechuic acid (>99 %), caffeic acid (>98 %), vanillic acid (>97 %), syringic acid (>95 %), ferulic acid (>99 %), kaempferol (>97 %), 1-1-diphenyl-2- picrylhydrazyl (DPPH), fluorescein and 2,2′-Azobis(2-amidinopropane) dihydrochloride (AAPH) were procured from Sigma Aldrich (USA). All the other chemicals and reagents were of analytical grade and purchased from Thermo Fisher Scientific (USA).

### 2.2 Plant material and preparation of plant extract

The mature fruit of *Stereospermum chelonoides* (L.f.) DC. was collected from natural habitat of Gandhmardan hills (Odisha, India) and the GPS coordinates were 20°54′06″ N, 82°49′17″ E at an elevation of 2428 m. The authentication of sample was done by Dr. Sharad Srivastava (Sr. Principal Scientist, Pharmacognosy Division, CSIR-NBRI, Lucknow, Uttar Pradesh, India) and was submitted to the institutional raw drug repository with collection voucher number (265420). The collected fruits were washed, chopped, shade dried, followed by oven drying at 40 °C and then pulverized to coarse powder.

5g of powdered sample was subjected to cold extraction separately in methanol, hydro-alcoholic (water: ethanol; 50:50 v/v) and water (50 mL), followed by continuous shaking for 6 h and stand at room temperature (25 °C ± 3) for 18 h [11]. The sample was filtered through Whatmann filter paper (No. 4) and the residue sample was re-suspended in fresh solvent for further extraction. The process was repeated thrice and the pooled filtrate was concentrated in rotatory evaporator (Buchi, Switzerland), concentrated extract was lyophilized (Labconco, USA) to solid residue and stored at 4 °C till future analysis.

### 2.3 Pharmacognostical analysis

The Pharmacognostical analysis of sample was done for quality assessment of sample through various parameters. Physicochemical parameters *viz*., ash values (total ash, acid insoluble and water soluble) and extractive values (hexane, alcohol and water soluble) were evaluated as per standard protocol mentioned in Ayurvedic Pharmacopoeia of India [11]. The phytochemical parameters *viz*., total sugar and starch, total phenolics and total flavonoid were also estimated through spectrophotometric method [13].

### 2.4 Nutritional analysis

#### 2.4.1 Proximate analysis

The air dried powder of *S. chelonoides* fruit were taken for proximate analysis. The samples were dried at 105 °C in the oven to determine moisture content [11]. Total protein was measured using Lowry method of protein estimation [14]. For lipid estimation, the samples were subjected to hot extraction using Soxhlet apparatus in petroleum ether followed by evaporation of solvent to yield lipids. The defatted sample left after lipid extraction was dried and ignited in muffle furnace to estimate the fibre contents [15]. Total carbohydrate content was estimated using the above determined factors in the following formula:

Carbohydrate (%) = 100 – [moisture (%) + protein percentage (%) + lipid (%) + ash contents (%)]

The total energy values of samples were determined using the formula:

K calories/100 g = 9 (crude fats (%)) + 4 (carbohydrates (%) + proteins (%)

#### 2.4.2 Elemental analysis

The dried and powdered fruit was digested through microwave digestion procedure (BERGHOF Speedwave-MWS-3+) using HNO3 (69 %, ACS quality, Germany). The total elemental content in the digested sample was determined by using Inductively Coupled Plasma Mass Spectrometry (ICP-MS Thermo iCAP TQ).

### 2.5 RP-HPLC-PDA determination of polyphenols

#### 2.5.1 Standard and sample solutions preparation

The standard solutions of gallic acid, protocatechuic acid, caffeic acid, vanillic acid, syringic acid, ferulic acid, quercetin and kaempferol was freshly prepared (1 mg/mL) in HPLC grade methanol, and stored at 4 °C while being prevented from daylight. Aliquots of the stock solution (standard) were diluted 10 times in a volumetric flask with HPLC grade methanol to prepare a working solution of 0.1 mg/mL prior to HPLC analysis. Five different dilutions were prepared for each individuals to evaluate limit of detection (LOD) and limit of quantification (LOQ). Samples for analysis were prepared by dissolving the 10 mg of lyophilized extract in their respective solvent (HPLC grade) to obtain a working concentration of 10 mg/mL. Working dilutions of standard and sample were filtered through 0.22 mm Millipore membrane filter (Pall, USA) for HPLC analysis.

#### 2.5.2 Instrumentation and chromatographic condition

The RP-HPLC-PDA quantification of phenolic compounds in fruit of *S. chelonoides* was operated on a high pressure liquid chromatography (HPLC) system by Waters (Massachusetts, USA), consisted of a pump for delivering the mobile phase (Waters-1525), auto sampler (Waters-2707) with a 30 μL loop for sample injection, photodiode array detector (Waters-2998) and column heater (Waters-1500) for maintaining the optimum temperature of column. All these units were operated and monitored on the Empower 3 Quick start software.

The separation of individual phenolic compounds were achieved on C_18_ RP-HPLC column (4.6 mm × 250 mm internal diameter, 5 μm particle size) (Massachusetts, USA), thermostated at 30 °C. The mobile phase consists of 0.01 M sodium acetate buffer (add glacial acetic acid until the pH reaches 3) (A) and acetonitrile (B). The mobile phase was eluted in isocratic manner (65:35 v/v; A:B) with the flow rate of 0.7 mL/min. The injection volume was 10 μL for each analyte and total run time was 30 min; detection wavelength was kept at 254 nm. Identification of peaks were done by comparing the retention time (R_t_) of standard peaks and the R_t_ of sample peaks, along with spectral information provided by photodiode array detector (PDA), operated over the range of 190–400 nm. The quantification was done based on linear regression curve of standard area vs. concentration.

### 2.6 In-vitro pharmacological assays

#### 2.6.1 Anti-oxidant activity

##### 2.6.1.1 DPPH radical-scavenging activity

The antioxidant activities of different extracts of *S. chelonoides* fruit were estimated by DPPH radical scavenging assay [16]. The IC_50_ value denotes the concentration of sample required for quenching 50 % of DPPH free radicals. The gallic acid was used as positive control.

#### 2.6.2 Oxygen radical absorbing capacity (ORAC)

The oxygen radical absorbing capacity (ORAC) of different extracts of *S. chelonoides* fruit was evaluated as per the method described by Prior et al. (2003) [17]. The net area under the curve (AUC) of the standard and samples were calculated. The ORAC value refers to the net protective area under the decay curve of the fluorescein in the presence of an antioxidant. The results were expressed as μmol Ascorbic acid equivalent (AAE) per gram of sample.

The antioxidant capacity (ORAC) equivalent to ascorbic acid was calculated as follows:

ORAC value = [(AUC_sample_ – AUC_black_)/(AUC_ascorbic acid_ – AUC_blank_)] [ascorbic acid] dilution factor.

#### 2.6.3 Antimicrobial activity

The antimicrobial activity of *S. chelonoides* fruit was examined by using agar well diffusion assay [18]. Briefly, different concentrations (5, 10, 15, 20 mg/mL) of fruit extracts was made-up in dimethylsulphoxide (DMSO). 100 μl of microbial suspension was swabbed and spread uniformly on the surface of freshly prepared Nutrient Agar for all the microbes. Thereafter, 6 mm diameter wells (as per the experimental design) were made-up with the help of sterile cork borer. Different concentrations of fruit extracts was aseptically poured in to the wells and left the plate in the laminar until the plant antimicrobials were completely diffused in to the well. Subsequently, the plates were incubated at 28 °C ± 2 for one week. All the experimental works were executed in triplicate. The diameter (in mm) of inhibition zones around the well was measured to evaluate the antimicrobial efficacy. Ciprofloxacin was used as positive control and DMSO is used as negative control.

### 2.7 Statistical analysis

Each observation was taken in triplicate and results were reported as mean ± standard deviation (SD). All the experimental observations were analyzed statistically by using SPSS version 18.0 software (SPSS Japan, Tokyo, Japan). The data were subjected to Tukey's multiple-comparison test to calculate the level of significance (p < 0.05) [19].

## 3 Results and discussion

### 3.1 Pharmacognostical analysis

Pharmacognostical analysis was done to establish the quality of the collected sample. Among the physicochemical parameters, total ash was highest, followed by water soluble and acid insoluble ash value. The water soluble extractive value was maximum followed by ethanol and hexane soluble extractives. The sample was extracted in different solvents on the basis of polarity gradient for evaluation of polyphenolic content and pharmacological activity, to know the best suited solvent for extraction.

Phytochemical analysis reveals that the fruit is rich in starch than sugar content. The hydro-alcoholic extract contained more phenolics and flavonoids than the water extract and methanolic extract (Table 1). As per our best knowledge, this is the first report on the pharmacognostical analysis of *S. chelonoides* fruit and can be utilized for quality control and standardization of samples.

**Table 1:** Pharmacognostical analysis of the fruit of Stereospermum chelonoides.

	Parameters		Sample*
	Total ash		3.60 ± 0.4
	Acid insoluble ash		0.24 ± 0.01
	Water soluble ash		3.45 ± 0.04
Physicochemical parameters		Hexane	0.022 ± 0.002
	Extractive values	Ethanol	0.21 ± 0.02
		Water	2.51 ± 0.02
Phytochemical parameters	Total sugar content		0.99 ± 0.01
	Total starch content		1.44 ± 0.04
	Total phenolic content	Methanolic extract	0.24 ± 0.01
		Hydro-alcoholic extract	2.26 ± 0.37
		Water extract	2.09 ± 0.083
	Total flavonoid content	Methanolic extract	0.087 ± 0.004
		Hydro-alcoholic extract	0.486 ± 0.01
		Water extract	0.372 ± 0.03

* Values are in %; ±standard deviation (SD).

### 3.2 Nutritional profile of fruit

#### 3.2.1 Proximate analysis

The proximate analysis of fruit suggests that it is a rich source of protein, lipid, and carbohydrates. The carbohydrate content in fruit is abundant i.e. (90.19 % ± 1.73) and will serve as a good source of energy 368.2 ± 3.94 Kcal/100g. In comparison to most of the utilized fruits, the fruit of *S. chelonoides* contains limited moisture content (4.07 % ± 0.02), indicating its high shelf-life in terms of microbial degradation [20]. The ash content is measure of the amount of inorganic minerals contained in the sample and results indicate *S. chelonoides* fruit is a richer source of minerals (2.58 % ± 0.04) than common utilized fruits like apple, litchi, mango and papaya [20]. Food fibers lower the risk of diabetes, heart ailments, hypertension and cholesterol and also help in increasing dietary bulk by easing the intestinal passage through absorption of water [21,22]. *S. chelonoides* fruit contains more crude fibers (3.46 % ± 0.02) than mango, papaya and litchi [20]. The Recommended Dietary Allowance (RDA) is 25–38 g/day [23] and the fibre content in the fruit may help in fulfilling the fiber RDA. The lipid content in the *S. chelonoides* fruit (0.75 % ± 0.01) is comparable to the amount found in the common fruits like apple, litchi, mango and papaya [20]. It lies in the range of >1 % and such low lipid range is sufficient to support the human fat needs and may also help to prevent cardiovascular diseases occurring due to high lipid consumption [24]. Proteins are the building as well as functioning material of the human body forming an integral part of cells, tissues, muscles and are involved in all metabolic processes in the form of enzymes, antibodies, etc. [25]. The fruit of *S. chelonoides* contains more protein (2.41 % ± 0.007) than commonly consumed fruits like apple, litchi, mango and papaya [20] and may serve as a low cost source of protein for rural and tribal communities. World Health Organization (WHO) recommends a daily intake of more than 400 g of fruit per person to protect against diet-related non-communicable diseases [26].

#### 3.2.2 Elemental analysis

The elemental analysis of *S. chelonoides* fruit is depicted in Table 2 and revealed that it is rich in micro-macro nutrients. The concentration of macronutrients Ca, Mg and Na in the fruit varied from 30.07 to 188.00 mg/100g. Calcium, apart from being an important building material for bones, also play a key role in body functions such as cell differentiation, neuro-motor activities, blood pressure maintenance, and various immunological responses [4,20]. The fruit of *S. chelonoides* is a rich source of calcium i.e., 84.78 ± 2.24 mg/100g which is higher than marketed fruits like apple, cashewnut, litchi, mango and papaya [20]. Magnesium is another element important for bones and acts as co-factor for more than 300 enzymes involved in various fundamental body processes such as myocardial contraction, glycemic and blood pressure control. The US Food and Nutrition Board suggests daily dose of 300–400 mg of magnesium for adults [27] and the fruit of *S. chelonoides* contains 188.00 ± 6.73 mg of magnesium per 100 g better than fruits like *Morus alba*, *Flacourtia indica*, *Syzygium caryophyllatum*, *Spondias pinnata* and *Phoenix loureirii* var. *pedunculata* [28]. The sodium content was found moderate i.e., 30.07 ± 1.89 mg/100g and is comparable to wild fruits *Elaecarpus tectorius, Semecarpus anacardium*, *Spondias pinnata* and less than *Morus alba*, *Flacourtia indica* and *Syzygium caryophyllatum* [25,29]. U. S. FDA suggests limited consumption of sodium is essential for the maintenance of body fluids and other functions, and high sodium content can cause high blood pressure and related heart diseases [30]. Thus, the availability of such macronutrients in *S. chelonoides* fruits validates its dietary usage and supports the need to enhance its commercialization and/or utilization in regular diet.

**Table 2:** Elemental analysis of the fruit of *Stereospermum chelonoides*.

Elements	Sample (mg/100g)*
**Calcium (Ca)**	84.78 ± 2.24
**Magnesium (Mg)**	188 ± 6.73
**Sodium (Na)**	30.07 ± 1.89
**Iron (Fe)**	49.10 ± 0.94
**Manganese (Mn)**	2.46 ± 1.05
**Zinc (Zn)**	0.90 ± 0.06
**Copper (Cu)**	0.56 ± 0.03

*n = 3; ±standard deviation (SD).

Micronutrients are dietary nutrients needed in very small amount but on deficiency, pose life threatening impacts on body health [31]. Iron is one of the most important micronutrient being integral part of hemoglobin and thus crucial for respiration. Iron deficiency, being the most common nutrient deficiency, leads to anemia and related complications such as reduced mental ability and diminished working capacity [25]. The fruit of *S. chelonoides* appears to be rich reservoir of iron i.e., 49.10 ± 0.94 mg/100g, better than most marketed fruits like *Punica granatum, Emblica officinalis*, *Phoenix dactylifera*, apple, mango, papaya and wild fruits *Aegle marmelos*, *Ficus carica*, *Morus alba*, *Annona squamosa* [20,25]. Other micronutrients Mn, Zn and Cu, were also found in fruit of *S. chelonoides* in the range of 0.5–2.5 mg/100g which is higher than most common fruits like apple, litchi, mango and wood apple [20]. These micronutrients are essential for proper functioning of the human body, with Mn being necessary for hemoglobin synthesis, Zn being co-factor for enzymes involded in nucleic acid metabolism as well as growth regulation and Cu being integral part of enzymes involved in metabolic reactions [20].

### 3.3 Quantification of bioactive polyphenols

**Fig. 1:**
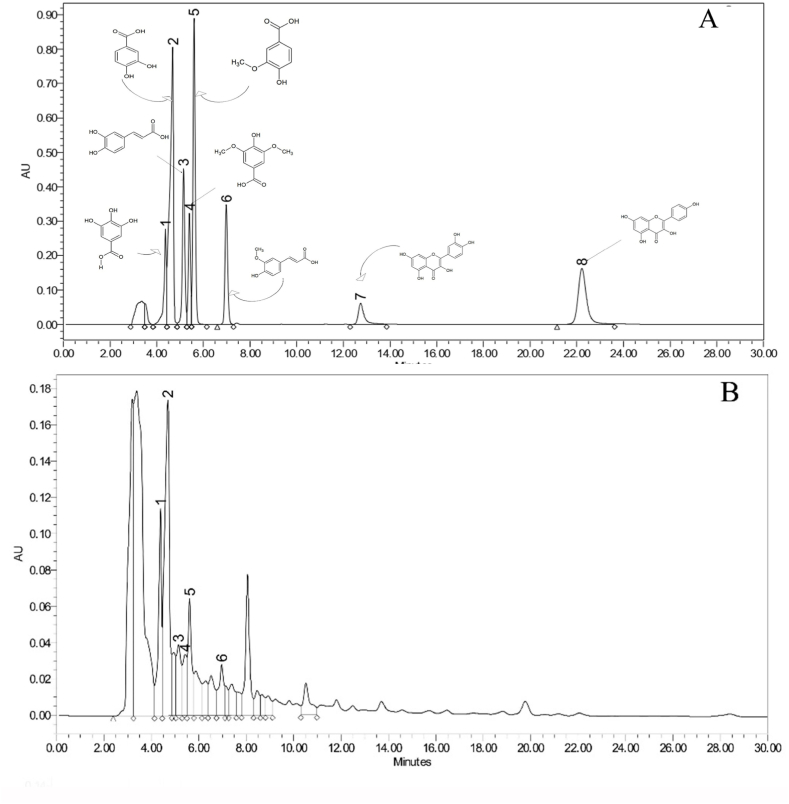

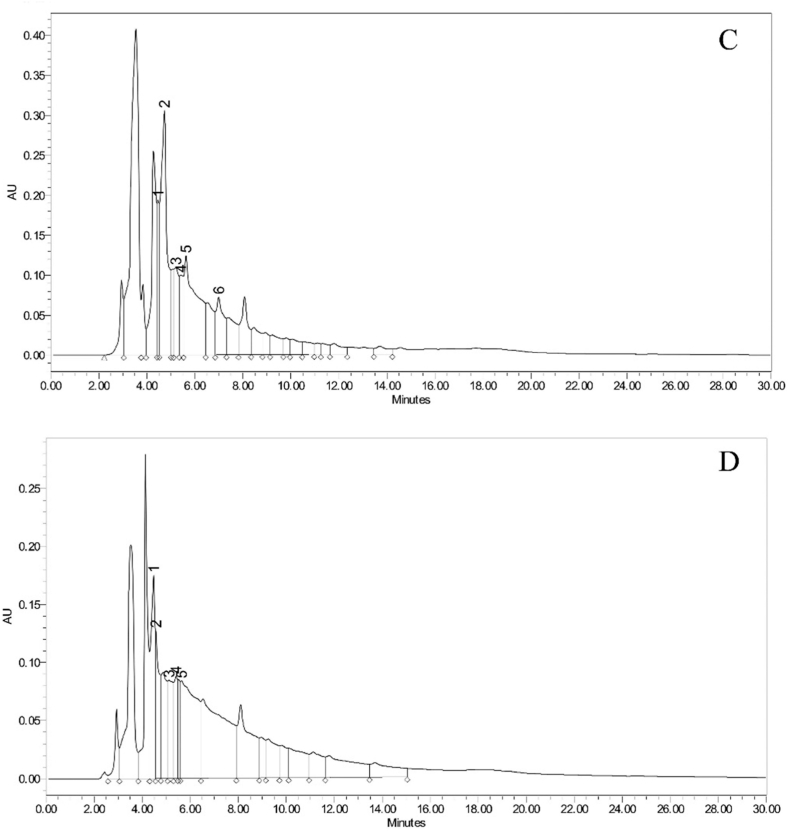
RP-HPLC-PDA chromatogram of a. standards mixture, b. methanolic extract, c. hydro-alcoholic extract and d. water extracts of *S. chelonoides* fruit. (1: Gallic acid, 2: Protocatechuic acid, 3: Caffeic acid, 4: Syringic acid, 5: Vanillic acid, 6: Ferulic acid, 7: Quercetin and 8: Kaempferol).

Polyphenols are the major component of food and have enormous health benefits. They exhibit potential antioxidant capacity and although they are not directly considered as nutrients, they are highly recommended in the diet in the form of fruits and vegetables. Hence, their quantification in targeted species will add value to the nutritional content of the fruit [32,33]. The method for the quantification of polyphenols was adopted from Misra et al. (2023) [34] and the calibration curve was plotted between standards area versus concentration using ICH guidelines [35]. The extractive yield of methanolic, hydro-alcoholic and water extracts are 4.8 %, 10.8 % and 12.4 % respectively. Eight polyphenols namely gallic acid, protocatechuic acid, caffeic acid, vanillic acid, syringic acid, ferulic acid, quercetin, and kaempferol were used as standards for quantification in the sample. All the standards were separated with retention time ranging between 4.373 min and 22.221 min (Fig. 1a). Out of eight, six compounds were detected in all the extracts in varying concentrations (Fig. 1b, c and d). Polyphenols were found in adequate amount in all the analyzed extracts (Table 3). Protocatechuic acid was found most abundant in both methanolic and hydro-alcoholic extracts, while water extract contained gallic acid in greatest amount. These values indicate the fruit to be rich source of polyphenolics, indicate its high nutraceutical potential as polyphenols attributed to have immense pharmacological properties such as antioxidant, antitumor, anti-inflammatory, antimicrobial, immunomodulatory, hepatoprotective, hypoglycemic, nephroprotective and neuroprotective activities [36,37].

**Table 3:** Quantification of phenolic acids in different extracts of the fruit of *S. chelonoides*.

Sr. No.	Compounds	Amount present in sample (μg/mL)*		
		Methanolic extract	Hydro-alcoholic extract	Water extract
1.	**Gallic acid**	60.94 ± 2.43	64.94 ± 3.29	120.84 ± 6.93
2.	**Protocatechuic acid**	65.15 ± 1.26	149.09 ± 7.83	35.51 ± 3.19
3.	**Caffeic acid**	18.02 ± 0.83	51.81 ± 2.41	42.53 ± 2.56
4.	**Syringic acid**	18.05 ± 0.59	47.50 ± 3.45	43.51 ± 3.23
5.	**Vanillic acid**	20.45 ± 1.03	131.28 ± 5.99	107.21 ± 5.21
6.	**Ferulic acid**	17.46 ± 0.53	65.02 ± 3.41	–

*n = 3**;** ± standard deviation (SD).

### 3.4 In-vitro pharmacological assays

#### 3.4.1 In-vitro antioxidant activity

**Fig. 2:**
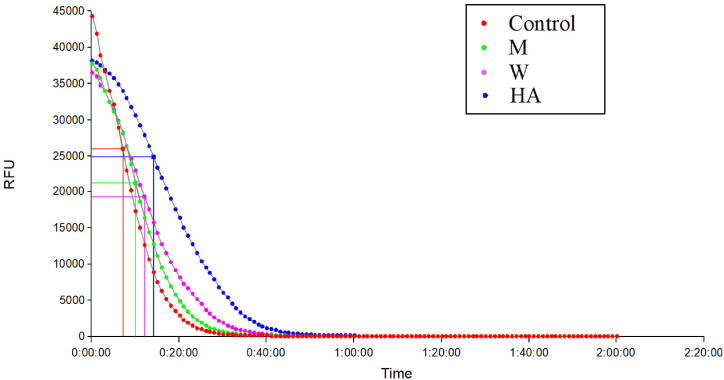
Fluorescein decay curve in presence of different extracts of *S. chelonoides* fruit. (M − methanolic, W- water and HA-hydro-alcoholic extract).

The DPPH radical inhibition was measured at five different concentration (20, 40, 60, 80 and 100 μg/mL) of methanolic, hydro-alcoholic, and water extracts and 1, 2, 3, 4 and 5 μg/mL of concentration is used for gallic acid. Inhibition of DPPH free radicals was observed in dose dependent manner as concentration of extract increases, percentage inhibition of free radicals increases. The anti-oxidant potential in terms of DPPH radical scavenging potential was found to be eminent in all extracts of *S. chelonoides* fruit (Table 4) with lowest IC_50_ value in hydro-alcoholic extract i.e., 42.86 ± 1.25 μg/mL followed by methanolic (63.96 ± 1.85 μg/mL) and water extracts (79.82 ± 3.19 μg/mL). The IC_50_ of Gallic acid was 2.09 ± 0.15 μg/mL (Fig. S2). The Oxygen Radical absorbing capacity (ORAC) determines the anti-oxidant potential of extracts in terms of ORAC values calculated using the fluorescein decay curve. The fluorescein decay curves recorded in the presence of *S. chelonoides* fruit extracts are depicted in Fig. 2. The ORAC value was found to be significantly higher for the hydro-alcoholic extract i.e. 3791.70 ± 95.86 μmol Ascorbic acid equivalents/gram (AAE/g), followed by water (538.94 ± 34.19 μmol AAE/g) and methanolic extracts (254.94 ± 24.73 μmol AAE/g).

**Table 4:** Anti-oxidant activity of the fruit of *Stereospermum chelonoides*.

Sample extracts		DPPH Radical Scavenging activity (IC_50_) ((μg/ml))	ORAC values* (μmol AAE/g)
Gallic acid		2.09 ± 0.15	–
*S. chelonoides*	Methanolic extract	63.96 ± 1.85	254.94 ± 24.73
	Hydro-alcoholic extract	42.86 ± 1.25	3791.70 ± 95.86
	Water extract	79.82 ± 3.19	538.94 ± 34.19

* AAE: Ascorbic acid equivalent.

Fruits are known to have rich antioxidant activity and the fruit of *S. chelonoides* also possesses immense antioxidant potential, similar to well-known fruits like *Spondias pinnata, Flacourtia indica*, *Syzygium caryophyllatum* [21,28]. The ability to scavenge free radicals might be due to the presence of polyphenols, found in the present study (Section 3.3). Research suggests oxidative stress plays a major role in several diseases such as cancer, rheumatoid arthritis, neurodegenerative diseases, cardiovascular diseases, kidney disorders, etc. [38] and antioxidant rich natural products may play a huge role in preventing these diseases.

### 3.5 Antimicrobial activity

The antagonistic activity of methanolic, hydro-alcoholic, and water extracts at different concentrations (5, 10, 15, 20 mg/mL) against *P*. *aeruginosa*, *V*. *cholerae*, *E. coli*, and *S*. *flexneri* were evaluated to examine the potential application of *S. chelonoides* fruit. The agar well diffusion plates clearly depicted that different extracts of *S. chelonoides* fruit significantly inhibit the growth of bacterial pathogens with variable potency. As evident from Table 5 & Fig. S1, hydro-alcoholic extract showed maximum inhibitory effect against *P. aeruginosa* with least IC_50_ value (19.26 μg/mL) as compare to other extracts. The methanolic extracts showed maximum intensity against *Vibrio cholerae* and showed maximum zone of inhibition (IC_50_ value 8.91 μg/mL). Whereas, hydro-alcoholic extract (IC_50_ 32.5 μg/mL) had significant results against *E. coli.* Similarly, hydro-alcoholic extract was the most effective extract which showed bactericidal activities against highly susceptible strain of typhoid namely *Shigella flexneri* and recorded 21.77 μg/mL IC_50_ value.

**Table 5:** Antimicrobial activity based IC_50_ values of *Stereospermum chelonoides* fruit against different bacterial pathogens.

IC_50_ (μg/ml)		*P. aeruginosa*	*V. cholerae*	*E. coli*	*S. flexneri*
Ciprofloxacin		4.85 ± 0.1	0.41 ± 0.04	0.11 ± 0.001	0.26 ± 0.02
*S. chelonoides*	Methanolic extract	24.64 ± 1.17^b^	8.91 ± 0.58^a^	–	25.20 ± 1.03^b^
	Hydro-alcoholic extract	19.26 ± 0.85^a^	16.50 ± 0.8^b^	32.50 ± 1.64^c^	21.77 ± 1.24^a^
	Water extract	38.06 ± 1.65^c^	–	–	33.50 ± 1.85^c^

*Means (n = 3) followed by the same letter(s) within the column are not signiﬁcantly diﬀerent according to Tukey's multiple comparison test (P < 0.05).

Earlier reports favour that leaf and stem of *Stereospermum* spp*.* possess significant (p < 0.05) inhibitory activity against wide range of food contaminants and human pathogens [39]. However, the similar property of *Stereospermum chelonoides* fruit was not explored yet. The previous reports on antimicrobial activities of different flora [40] demonstrate that, inhibitory effects of plants are because of the enormous reservoir of bioactive molecules including phenols, flavonoids, alkaloids, saponins, terpenoids, etc. [41,42]. The resulting antimicrobial activity of *S. chelonoides* fruit might be due to the phenolic acids as quantified in Section 3.3. The antagonistic activity of *S. chelonoides* fruit proves consumption of this fruit will help in preventing the disease caused by above mentioned bacteria and will also facilitate storage (low food spoilage).

## 4 Conclusion

The study concludes that *S. chelonoides* fruit, has immense dietary potential having high proximate values, and elemental composition. The study establishes the pharmacological potential of the species with several bioactive phenolics and have promising antioxidant activity along with antimicrobial action, against common human pathogens. The study favours the consumption of *S. chelonoides* as a dietary supplement for local inhabitants, however, *in-vivo* safety and toxicity studies are much-needed requisites before promoting the nutraceutical usage of the fruit for its human consumption.

## Data availability statement

Data included in article/figures/tables/supplementary material.

## CRediT authorship contribution statement

**Mridul Kant Chaudhary:** Writing – original draft, Methodology, Investigation, Formal analysis. **Deepali Tripathi:** Writing – original draft, Methodology, Formal analysis. **Ankita Misra:** Writing – review & editing, Validation, Formal analysis. **Satyendra Pratap Singh:** Visualization, Formal analysis. **Pankaj Kumar Srivastava:** Writing – review & editing, Formal analysis. **Vartika Gupta:** Investigation, Formal analysis. **Rabinarayan Acharya:** Writing – review & editing, Resources. **Sharad Srivastava:** Writing – review & editing, Supervision, Conceptualization.

## Declaration of competing interest

The authors declare that they have no known competing financial interests or personal relationships that could have appeared to influence the work reported in this paper.
