# Supplementary material for: Nutritional characteristics of Stereospermum chelonoides (L.f.) DC., an underutilized edible wild fruit of dietary interest
Source: Heliyon. 2024 Jan 11;10(2):e24193. doi: 10.1016/j.heliyon.2024.e24193 (PMC10826151; doi:10.1016/j.heliyon.2024.e24193)
Supplement: Multimedia component 1 [file mmc1.doc]

**Nutritional characteristics of Stereospermum chelonoides (L.f.) DC., an underutilized edible wild fruit of dietary interest**

Mridul Kant Chaudharya!,Deepali Tripathia1!,Ankita Misraa, Satyendra Pratap Singha2, Pankaj Kumar Srivastavab , Vartika Gupta­­­b, Rabinarayan Acharyac and Sharad Srivastavaa*

aPharmacognosy Division, CSIR-National Botanical Research Institute, Lucknow (U.P.), 226001, India

bEnvironmental Technologies Division & ENVIS – NBRI, CSIR-National Botanical Research Institute, Lucknow (U.P.), 226001, India.

cDirector General, CCRAS, New Delhi, 110058, India.

1Present Address: FEST division, CSIR-Indian Institute of Toxicology Research, Lucknow (U.P.), 226001, India.

2Present Address: Department of Postharvest Science, Agricultural Research Organization, Volcani Center, P.O.B 15159, HaMaccabim Road 68, Rishon LeZion 7505101, Israel.

!Authors contributed equally

*Corresponding author: Dr. Sharad Srivastava, Chief Scientist and Head, Pharmacognosy Division, CSIR-National Botanical Research Institute. E-mail address: sharad_ks2003@yahoo.com Telephone number: +91-0522 2297818

**Figure caption**

**Fig. S1.** The inhibitory activity of different extracts of *S. chelonoides* fruit at different concentrations against *Pseudomonas aeruginosa*, *Vibrio cholerae*, *E. coli*, and *Shigella flexneri*. (1: 5 mg/mL, 2: 10 mg/mL, 3: 15 mg/mL, 4: 20 mg/mL).

**Fig. S2**. Calibration curve of gallic acid (A) and test extract(s), methanolic extract (B), hydro-alcoholic extract (C) and water extract (D) against DPPH radical scavenging assay at five different dilutions.


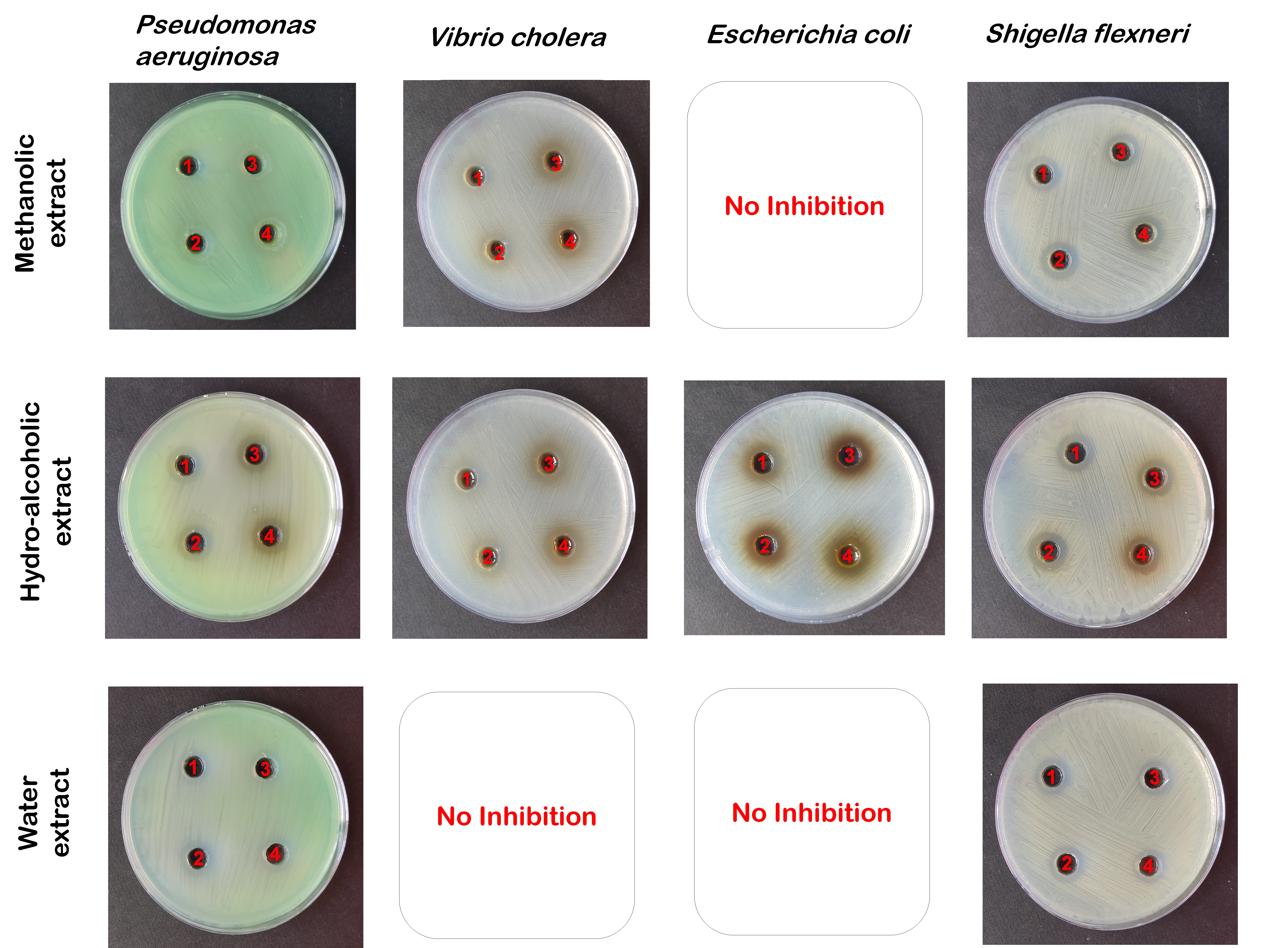


**Fig. S1.**

B

A

C

D


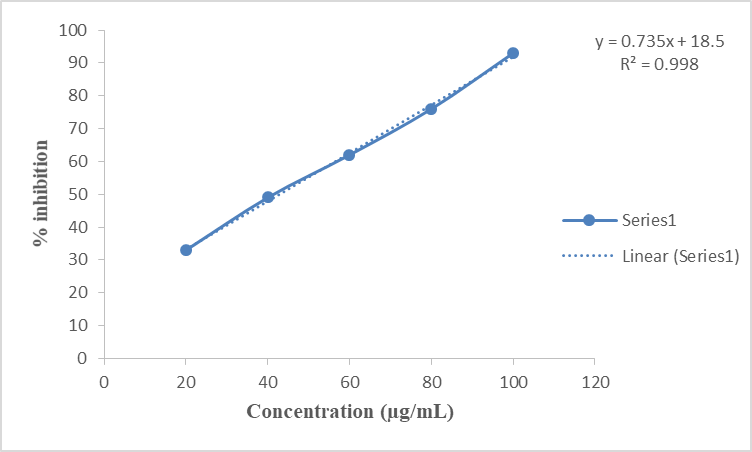

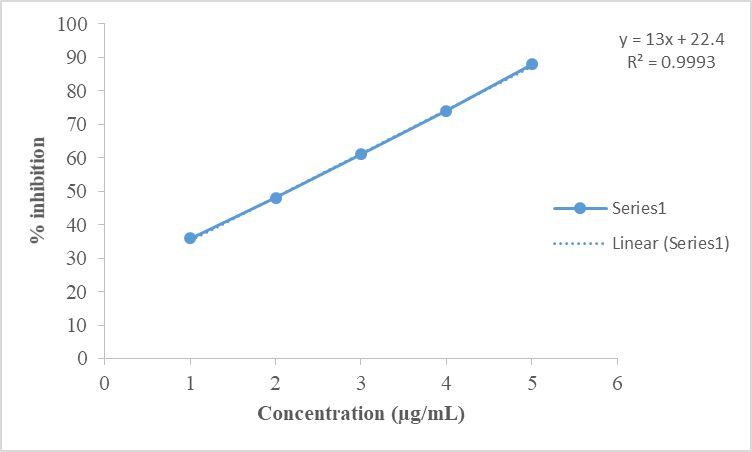

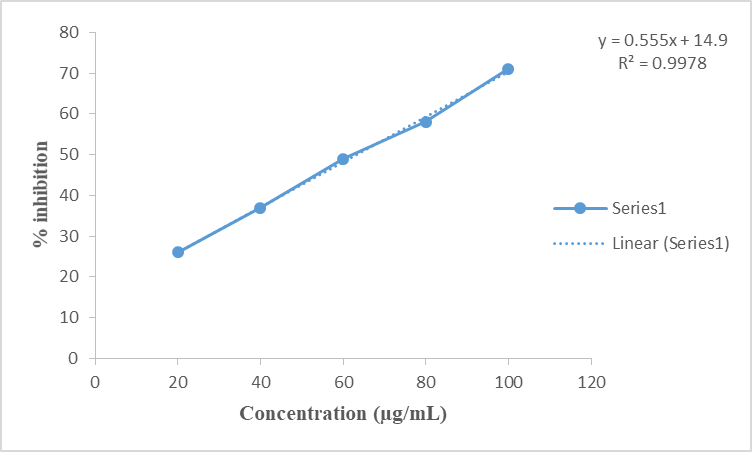

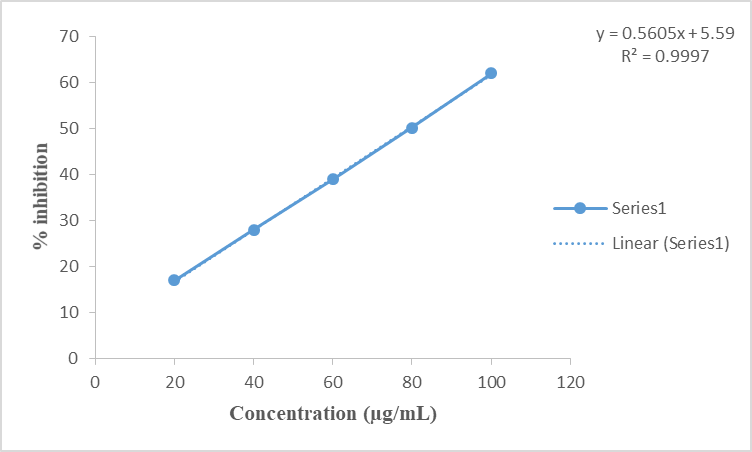


**Fig. S2.**
